# Some behavioral aspects of energy descent: how a biophysical psychology might help people transition through the lean times ahead

**DOI:** 10.3389/fpsyg.2014.01255

**Published:** 2014-11-03

**Authors:** Raymond De Young

**Affiliations:** Environmental Psychology Lab, School of Natural Resources and Environment, University of MichiganAnn Arbor, MI, USA

**Keywords:** biophysical psychology, environmental psychology, conservation psychology, energy descent, behavior change, prefamiliarization, embedded benefits, psychological well-being

## Abstract

We may soon face biophysical limits to perpetual growth. Energy supplies may tighten and then begin a long slow descent while defensive expenditures rise to address problems caused by past resource consumption. The outcome may be significant changes in daily routines at the individual and community level. It is difficult to know when this scenario might begin to unfold but it clearly would constitute a new behavioral context, one that the behavioral sciences least attends to. Even if one posits a less dramatic scenario, people may still need to make many urgent and perhaps unsettling transitions. And while a robust response would be needed, it is not at all clear what should be the details of that response. Since it is likely that no single response will fix things everywhere, for all people or for all time, it would be useful to conduct many social experiments. Indeed, a culture of small experiments should be fostered which, at the individual and small group level, can be described as behavioral entrepreneurship. This may have begun, hidden in plain sight, but more social experiments are needed. To be of help, it may be useful to both package behavioral insights in a way that is practitioner-oriented and grounded in biophysical trends and to propose a few key questions that need attention. This paper begins the process of developing a biophysical psychology, incomplete as it is at this early stage.

## INTRODUCTION

Since its beginning, the environmental movement has documented the declining state of the world. Stark language is used to catalog the many threats to the planet’s natural systems and to the human settlements that depend on those systems. These deeply pessimistic outlooks continue to proliferate despite the fact that, when used alone, such accounts almost never increase concern or motivate action. Thankfully, there are also published inspirational stories—case studies of responses to environmental problems (Hertsgaard, 1999). There is no better antidote to social and environmental pessimism than Hawken’s (2008) *Blessed Unrest*. Yet, if either the gloomy or the inspirational efforts had their desired effect then it might not be necessary to keep publishing quite so much on the topic.

This seems to call for a frank reassessment of our prospects starting with an update on the current context (Benson and Craig, 2014). The idea that context matters has long been embedded in the social sciences (Sommers, 2011). Yet, the changing biophysical context of everyday behavior has received little attention. It is not the goal of this paper to present another catalog of the downward spiral or to provide a selection of rousing success stories, although the importance of the latter is well-established. The intent is to show how a slowly changing biophysical context may be causing an entirely new behavioral context to emerge. Furthermore, the intended audience is not the general public but the many behavioral scientists and environmental practitioners who strive to better the world. The paper ends with several questions that might help direct research aimed at responding to the new behavioral context. From such research might emerge new approaches to helping people to live well while they live within ecological limits.

## THE NEW BIOPHYSICAL CONTEXT

How ever vast were the resources used to create industrial civilization, they were never limitless. Biophysical constraints, always a part of human existence, could be ignored during these past few centuries, a unique era of resource abundance. This is no longer possible.

Many of the difficulties now being faced have their origins in a centuries-long consumption and construction binge and, soon, in its abrupt culmination. Both individual and collective behaviors have spawned multiple challenges, each now beginning to weaken industrial civilization. This is the premise of McKibben’s (2010) book *Eaarth*. The world onto which we were born has been so disrupted that it’s not the world on which we now live. McKibben (2010, p. 33) points out that, “We know, definitively, that the old planet “worked.” That is, it produced and sustained a modern civilization. We don’t know that about the new one.”

These difficulties are manifestations of Harris’s (1979) theory of cultural materialism which provides a scientific explanation for the ecological origins of civilization. Harris argues for the primacy of society’s relationship with its environment—what he labels infrastructure and includes all forms of provisioning and supporting of daily life. Heinberg (2014, p. 5), in a superb synopsis, applies Harris’ theory to emerging environmental circumstances and suggests that, “… we probably face an infrastructural transformation at least as significant as the Industrial Revolution.” The reason why western industrial society may be on the verge of a major transition can be appreciated by reviewing the current natural resource and environmental situation.

### ENERGY DESCENT

Western society may be challenged by the declining rate of extraction of global energy resources (Bardi, 2014) and in particular the liquid fossil fuels that are the lifeblood of industrial civilization (Hirsch et al., 2005; Princen et al., in press). The fossil fuels era, that period when fossil fuels came to dominate all other energy sources, began in the United states and worldwide as recently as the 1890s (Smil, 2011, 2012). This era is bounded by two unassailable facts—the planet’s carbon stores are finite and the exponential growth in the use of these resources is unsustainable (Hubbert, 1996). But a sometimes overlooked issue here is not how much of a resource was once in place or now remains; but the geophysical limit on its rate of extraction.

For each reservoir of liquid fossil fuel, a maximum rate of extraction is eventually reached after which production plateaus, before an immutable decline. This limit to the rate of extraction is a matter of geology, not economics or technology or policy, a fact long understood by geologists but little appreciated by others. The maximum rate of oil production in the United States occurred in 1970 (Heinberg, 2012). The global on-shore peak occurred in 1984, Alaska’s North Slope in 1989 and the North Sea in 1999. Over 60% of oil-producing countries have peaked or plateaued their rate of petroleum production.

One point of clarification about peak production is that it refers to the rate of extraction; it has nothing to do with the volume of the planet’s energy stocks. And thus, despite a great deal of misinformation and misunderstanding, it is not about suddenly running out of fossil fuels. The peak in the production rate of petroleum typically happens near the point where half a reservoir’s resource still remains. Given their enormous volume, fossil fuels will likely be extracted from the Earth’s crust for years to come; what will decline is the amount available to society over any given time period. The concern here is the downslope from the peak rate of production, a psychologically unchartered territory.

Fortunately, the decline in the rate of production is often a slow, prolonged process. In the past, when a particular reservoir’s production rate peaked, this slow decline provided the time needed to transition to new sources and develop new technologies. Previously, this adjustment occurred seamlessly, behind the scenes, as a normal part of the hydrocarbon exploration and development process. However, recently, as more reservoirs pass their peak extraction rate, this adjustment has become difficult, with one manifestation being the end of cheap energy (Campbell and Laherrère, 1998). Apparently, there are fewer high-quality sources whose production rate has not yet peaked. Now, the transition might need to be to realistic expectations and new patterns of end-use behavior.

The dynamics of the peaking of production rates at the global scale and the consequences of descending from that peak are being vigorously debated, albeit by a relatively small number of experts (Maugeri, 2004; Heinberg, 2007a,b; Hall and Day, 2009). The economic and political vulnerabilities are important (Kerschner et al., 2013) but so too are the social and psychological dynamics, yet the latter are only rarely explored (Frumkin et al., 2009; Friedrichs, 2010; Lambert and Lambert, 2011; Neff et al., 2011; Butler et al., 2012; De Young and Princen, 2012). The high emotions and huge stakes that play out in this debate (as well as the eerie political and media silence) make it hard for individuals and communities to form a coherent understanding or a timely behavioral response.

However, slowly, an agreement is emerging: industrial society is leaving the era of cheap and abundant energy; it will not return to it but for short respites. The global production rate of liquid fossil fuels soon may begin, or is already beginning, a drawn-out leveling and then slow descent, with other fuels and materials soon to follow the same pattern. Then industrial civilization, having already scoured the planet of new sources, will experience biophysical limits as a steady headwind against which it must labor. At that point will start, as Klare (2012) writes, a race for the little that is left. Indeed, that race may have already begun (IEA, 2014).

Thus, it might seem that a lively public discussion about the dynamics and timing of energy descent should be initiated. Yet, from a psychological point of view, exactly when these events begin is much less important than acknowledging that they will occur. In fact, given how essential material resources, and in particular energy flows, are to the smooth functioning of modern society, debating the exact timing might become a dangerous distraction. Certainly western nations should understand and acknowledge the biophysical limits they face but rather than dwell on the causes, it might be more useful to invoke the precautionary principle—what was previously referred to as common sense (i.e., a responsibility to help society protect itself from exposure to harm when systematic investigation identifies a plausible risk). The task would then be to construct and test affirmative responses to unfolding resource constraints.

### NET ENERGY

It takes energy to get energy and transform it into socially usable forms. Maintaining a sufficient net surplus is what has become harder to do. This is due to declines in an ecological metric referred to as energy returned on energy invested (EROEI, sometimes EROI). This notion, also referred to as net energy, is the ratio of the amount of usable energy society acquires from a particular energy resource to the amount of energy it expends to obtain that energy. EROEI was first studied as the concept of net energy by White (1959), and particularly Odum (1973). It was developed by Hall (1972) in a biological context and later applied to the study of biophysical economics (Cleveland et al., 1984) and energy systems (Cleveland, 2005; Hall, 2011, 2012).

Energy returned on energy invested is hardly ever used in policy-making, dominated as the latter is by economistic reasoning. This history, and the simplicity of the concept itself, should not distract from its profound implications and future utility. The metaphor of low-hanging fruit can be used to explain the notion from a decision-making perspective. Extraction occurs first at the more attractive opportunities in easy to reach, hospitable regions containing high-quality resources (e.g., uncontaminated, high density) near the surface which thus makes them easy to extract, process and transport. Later, the resources are sought in inhospitable locations (e.g., offshore, deep-water, arctic, dangerous) and are of lower quality, thus becoming much harder to recover, refine and deliver. Harder here means the need to employ more equipment, larger systems and complicated logistics, all of which themselves consume energy during their lifecycle. For instance, many of the newer unconventional energy sources require advanced technologies, massive capital investment and, most significantly, increased amounts of energy which from a thermodynamic perspective is the ultimate determinant of net energy availability.

This pattern—pursue the easiest to get first and the more difficult to obtain later—while a perfectly sensible decision economically and technologically, means that over time the net energy available to society inevitably declines. Perhaps this would be nothing more than an interesting story for technocrats except that *now is later*. We are at the part of the story where there is less and less net energy with which to operate society. Political scientist Homer-Dixon (2006) argues that just such a declining EROEI was one factor underlying the collapse of the Western Roman Empire in the fifth century CE. Tainter (1988) presents the complementary idea of diminishing marginal returns of societal complexity to explain the decline of that and numerous other civilizations, and then goes on to apply this concept to contemporary civilization (Tainter, 2000, 2006, 2011, 2013).

At the beginning of the petroleum era the EROEI ratio was extremely high. The initial massive surplus of net energy may have misled society with the false prospect of endless physical growth. False because, while it went unnoticed, net energy has been on a relentless decline (Hall, 2012). Certainly, when the EROEI ratio of an energy source is less than one then that source becomes an energy sink and is useless to society as a means of maintenance, let alone growth. That a vast store of the Earth’s fossil carbon could become an energy sink is not an outlandish idea. Heinberg (2007b) reports that a large amount of the coal remaining underground will require more energy to extract than it will produce when burnt, thus becoming an energy sink rather than an energy source.

The question of what minimum EROEI is needed to support a complex society has been considered by Hall et al. (2009, 2014), Guilford et al. (2011), Hall (2011, 2012), Lambert and Lambert (2011), and Lambert et al. (2013, 2014). In this analysis it matters tremendously what features of society are believed to be necessary or desirable. As the services included in the definition increase, so too does the EROEI ratio needed to support that society.

Most often the EROEI ratio is calculated upstream at the well- or mine-head and includes only those energy costs directly related to the hydrocarbon exploration and production process. In an effort to make the EROEI concept more useful for social decision-making, Hall et al. (2009) and Hall and Klitgaard (2012) have expanded the methodology to also account for the many indirect energy costs experienced when providing services to society (e.g., the energy costs of transport, infrastructure, manufacturing, provisioning, maintenance). This is the net energy downstream, near the point of end-user consumption, and is reported as the extended-EROEI ratio.

A 3:1 extended-EROEI ratio is considered the “bare minimum for civilization” (Hall et al., 2009, p. 45; Hall and Klitgaard, 2012, pp. 318–319). Although at such a level modern society would lack the surplus energy needed to support most of the higher-level services that it has come to expect. In Western society, more than the most basic provisioning would require an extended-EROEI ratio of about 7 or 8:1, adding basic education would require a ratio closer to 9 or 10:1, health care and higher education would require a ratio near 12:1 and the arts and other noble pursuits push the required ratio even higher (Hall and Klitgaard, 2012; Lambert et al., 2013). Dadeby (2012) applied the extended-EROEI methodology to global production data and estimates that the current global ratio is at 6:1. Equally concerning is that most alternative, unconventional and biologically based sources of energy also have very low ratios.

A similar analysis can be applied to food production. The United States food system, as a result of the juggernaut of industrialization, consumes the vast majority of the energy it utilizes in post-farm processing, distribution and household preparation. Based on 2002 USDA data, the United States now invests over 12 calories of energy for each calorie of food consumed once waste and spoilage are accounted for. Of these, only 1.6 calories are used on the farm (Canning et al., 2010). This analysis suggests that industrial food production is unsustainable without fossil fuel inputs.

As the overall EROEI declines, society has less and less net energy to use for its needs as an ever-increasing percentage of the extracted energy is consumed by the hydrocarbon exploration and production industry itself. The day may be approaching when the surplus net energy from conventional and unconventional hydrocarbon sources becomes insufficient for maintaining, let alone building out, complex industrial society or the much hoped for renewable energy systems. Furthermore, if there is not enough surplus net energy to respond effectively to the many environmental issues being faced then a reallocation away from discretionary uses may be unavoidable. Taken together, this poses a psychological challenge: how might people, individually and collectively, respond to a substantial decline in discretionary resource use?

### TECHNOLOGY

It is here that technology is commonly invoked as a solution. It has become a habit to celebrate modern technical ingenuity, and in fact its application has produced innovations that have propelled dramatic and absolute increases in material well-being. This leads many people to be extremely optimistic, even complacent, about finding technological solutions to all problems challenging society. This is a dominant outlook, what has been called the industrial progressive worldview (Princen et al., in press). But there is reason to be less optimistic and certainly not complacent (Costanza, 2000; Alexander, 2014).

The great bulk of inventiveness has been aimed at increasing efficiency in all the many stages of material and energy extraction, conversion, use and reuse. But initial efficiency gains are the easiest to attain—they are the proverbial low-hanging fruit—after which the law of diminishing returns makes further improvements ever more difficult to achieve. Butler et al. (2012) point out that while efficiency-driven approaches have great potential they can never keep up with exponential growth in the use of materials or energy.

There is a behavioral side story here. Some energy-efficient technologies have paradoxically contributed to an increase in resource use (e.g., energy-efficient light bulbs actually increase electrical consumption when it seems justified to install them in greater numbers to provide increased illumination). This rebound effect is well documented (Hofstetter et al., 2006; Hanley et al., 2009; Madlener and Alcott, 2009; Norgard, 2009; Otto et al., 2014) but Princen (2005) has expanded on its implications by explaining how efficiency-driven modernization has misled us, and calling for a shift toward a logic of sufficiency.

Overall, despite a century of technological progress that provided noteworthy efficiency gains and innovations in design, policy and practice, there has been not the expected decrease but an absolute increase in aggregate natural resource consumption (Princen, 2005). Whether this is incorrectly attributed solely to population growth, or it is correctly noted that per capita consumption has also grown, the negative environmental outcomes remain unchanged. Society may be experiencing the unwelcome consequences of having used its technological ingenuity to attempt limitless growth on a finite planet.

But there is another and more fundamental reason to worry about whether technology can be an aid in dealing with energy descent. The technological foundation for an industrial society (e.g., logic and mathematics, physics, chemistry, engineering) and even the demonstration of advanced concepts and devices all existed for a great many centuries. But the available energy sources of muscle, biomass, water- and wind-power were unable to provide the energy quantity or quality needed to support an industrial revolution. It took the discovery and large-scale extraction of fossil fuel energy to enable the building of an industrial civilization. Simply put, the process of technological modernization was energy limited not knowledge limited. As Greer (2012, p. 97) points out, “It’s as arrogant as it is silly to insist that people in past ages weren’t as resourceful and ingenious as we are.” Thus, while technical cleverness is credited with creating a vibrant industrial society, and it is hoped that its continued application will solve all forthcoming problems, history suggests that it was instead a one-time gift of resource abundance that got us here. After all, inspired technologies cannot create energy and industrial prowess cannot negate the laws of thermodynamics, they can only transform finite energy and material resources into forms useful to society.

Nonetheless, boosters will claim that new technologies are unleashing vast amounts of energy from new sources, as, for instance the assertion that the recent innovation of hydraulic fracturing is releasing natural gas and light tight oil from new formations. What may go unnoticed is that neither the technology nor the low-permeability formations being mentioned are new; both have been long known to the hydrocarbon industry. What make feasible such extraction are the high fossil fuel prices that allow the use of previously prohibitively expensive technologies. But even this process of alchemic transformation may reach a limit. Research suggests that, to sustain urban growth, technical and social innovations must emerge at a continually accelerating rate (Bettencourt et al., 2007). Yet, requiring that individuals and organizations invent and adapt at an exponential rate in order to avoid stagnation and eventual collapse quickly reaches a limit; exponential growth in any process is, after all, unsustainable.

The end of resource abundance may require that techno-optimism be tempered since energy descent will make the effectiveness of technology that much lower. And while technical skill will be frequently called upon, the most that might be expected from it is to slow the approach of a resource-limited future; technology cannot fundamentally change that outcome.

## THE NEW BEHAVIORAL CONTEXT

Previously, when growth was an easy thing to do, it was possible to disregard the biophysical foundation of civilization. During a relatively brief period of material aﬄuence behavioral scientists could focus on social and psychological needs unhindered by natural resource constraints. Later, as ecological limits were first anticipated (Meadows et al., 1972) and then became apparent (Meadows et al., 2004; Bardi, 2011), some among us advocated for the study of conservation behavior (Daly, 1977; Stern and Kirkpatrick, 1977; Henion and Kinnear, 1979; Cone and Hayes, 1980; Cook and Berrenberg, 1981; Stern and Gardner, 1981). This was followed by decades of environmental and conservation psychology research that has led to an explosion of insights on theories of social change and intervention guidelines for promoting environmental stewardship behavior (Clayton and Myers, 2009; Clayton, 2012; De Young, 2013).

Earlier, it seemed that the major task facing modern society was to create a shared vision of a sustainable society providing a permanent prosperity lived within ecological constraints (Costanza, 2000). The changing biophysical context may foreshadow a less optimistic outcome and it certainly foreshadows a more difficult social transition. One reason for this is that the earlier behavioral context assumed that a consumer-focused, industrial society *could* be made sustainable. The observations above—that the rate of energy and material production may soon plateau and then decline, and that technological innovation may help ease a societal transition but will not eliminate the need for one—brings that prospect into question. It is at least conceivable that society soon will face the biophysical situation just outlined and need to learn to function within a newer, perhaps more austere, behavioral context. In an effort to aid this transition it might be useful to merge insights from across the social sciences into a biophysical psychology.

### A TRANSITION, NOT A PROBLEM SOLVED

Re-emerging biophysical constraints are not problems, at least not in the normal definition of that word. They are complex ecological predicaments, unsolvable situations that likely will play out over the rest of this century and the next. As Greer (2008) points out, we approach a problem by looking for a solution, if one is found and can be made to work then the problem is solved. In contrast, complex predicaments do not have solutions, they must be endured (Smith, 2014). Certainly, in order to thrive, we must acknowledge and respond to them, but even an effective response does not make the predicament go away. The response does not alter but rather accommodates the new reality. This is adaptation in its classic usage: to recast or change behavior patterns into new forms so as to fit the new ecological situation.

Furthermore, biophysical limits present behavioral challenges that are unlike those of emergencies and crises. The needed responses might be better characterized as transitions, processes that differ from an emergency or crisis in the depth of change required, the time frame involved and the prospects about the future (Princen, 2014). An emergency is immediate, with a central precipitating event calling for a rapid and focused reaction designed to restore life to the condition before the emergency. A fire consumes, prompting reaction and rebuilding, after which life resumes as before. Likewise, a crisis unfolds over months and years, and although it may resist a complete resolution, with recovery and remediation there is eventually a return to business-as-usual. The oil shocks in the 1970s created a crisis among the industrialized nations, stalling consumer spending and thus economic growth. But with new policies and behaviors (e.g., strategic petroleum reserves, residential energy conservation) and a shift to other energy sources, industrial activity ever-so-slowly returned to growth.

It may be possible to analyze an energy descent as a crisis, at least in its very early stages. Lambert and Lambert (2011) have anticipated a crisis response by the American people to declining EROEI by examining the resulting individual and social-system stress as well as the coping mechanisms employed and the resulting institutional effects (see also Hobföll, 1989). Then again, in a transition, the trajectory may be fundamentally different. The triggering issues are likely complex and may involve multiple interacting events (i.e., simultaneous geopolitical instability, energy descent and climate disruption). The effects may be broadly spread over physical and social systems, very slow to emerge and even slower to be widely acknowledged. The resulting changes, and the response to them, can span decades, entire lifetimes, even centuries. Suffice it to say that in an emergency or crisis the intention is to weather the storm and “get back to normal.” Whereas in transition it is understood by the leaders and public alike that there is no possibility of going back, the intent is to “get to a new normal.” Behaviors in an emergency or crisis are reaction and recovery; in transition they are innovation and adaptation.

Moreover, in a transition, the responses likely would need to be maintained and periodically updated over a lengthy period. Unfortunately, society has little familiarity with the long-drawn-out planning and management needed to respond well to biophysical limits. For while social institutions and individual behaviors exist for handling a comparatively brief emergency or crisis, and while changes that occur exceedingly slowly over a period of centuries rightfully can be left to the process of normal social adjustment, there exists little guidance for the mid-range, a many decade- or century-long transition. Parts of a science of such mid-range transformations are being developed. For instance, a psychology of prospection is emerging and will be useful to long-drawn transitions (Seligman et al., 2013). Navigating long timeframes can be difficult. The effects of behaviors changed today might only be appreciated in 80 years’ time. This lag has the potential to undermine motivation. Or, in a more positive framing, we must understand the conditions under which long-term planning is possible (Princen, 2009) and discover those situations where a lag between cause and effect does not undermine motivation but, perhaps unexpectedly, strengthens it.

Such an extended behavioral timeframe is inherent in the wedges concept proposed for keeping carbon emissions in check. Here, instead of searching for a single large-scale solution, Pacala and Socolow (2004) note that a number of smaller options already exist for reducing our collective environmental impact. They propose breaking the required changes down into manageable wedges each addressed by a different existing technology or policy. This idea has a behavioral version whereby over one third of the needed reductions can be accomplished by currently understood changes in everyday household activities (Dietz et al., 2009).

Yet, when viewed as a century-long process of behavior change, two significant issues emerge. The technological and behavioral wedges adopted early on must stay adopted, perhaps difficult in a world seemingly addicted to frenetic change and social reinvention. This needed durability presents an intervention challenge since the behavioral sciences are only starting to understand how to initiate robust self-sustaining and/or easily restarted behavior change (De Young, 2000, 2011; Abrahamse et al., 2005; Werner, 2013). Equally challenging is that to stabilize the positive outcomes, each wedge adopted must expand over time. Since the early changes do not solve the problem, there is a need to constantly innovate and adopt new behaviors and policies. While the near-term changes involve the adoption of currently known approaches (e.g., green consumer behaviors, efficiency-focused policies), changes a few decades hence can scarcely be imagined. Behavioral scientists acknowledge this difficulty by stating that later on, “[l]ifestyle changes may become necessary in the out-years under constrained energy supply or economic growth scenarios …” (Dietz et al., 2009, p. 18455).

But to label these needed future responses as “lifestyle changes” may mislead in two ways. First, the term itself would seem to imply that gentle and slight changes in daily habits will suffice. Yet, shifting consumer and living habits toward more green choices might prove to be a totally insufficient response should the biophysical events unfold as outlined above. Second, near the end of this century, day-to-day behavior patterns will need to consume nearly an order-of-magnitude less energy and materials than are currently used. The environmental movement has previously argued for major reductions in resource consumption but rarely have changes of this magnitude been envisioned.

It is not at all clear that the general outline, let alone the details, of these future behaviors are now known. It is difficult to imagine what daily life might be like after such a drastic reduction in resource consumption. Certainly it is possible to live at such a low-energy and material flux, indeed almost all of human history occurred within a pre-industrial low-energy context. But what is not known is how to live at such an austere level while still enjoying the comforts and conveniences afforded by industrial society. Frankly, it may not be possible for members of Western societies to maintain anything close to a contemporary life pattern while also living within the new biophysical context. If this proves to be the case, then the responsibility of behavioral scientists is to now explore and then prepare people for the social and psychological implications of that realization. At a most basic level, many people will grieve from losing an aﬄuent lifestyle or from losing the belief that material growth will one day provide for such an existence. If nothing else can be accomplished, then it will be praiseworthy to help people cope throughout this transition, to help them to function better than they would otherwise.

There is interesting work along this line that explores the effects of energy descent on public health (Frumkin et al., 2009; Neff et al., 2011; Poland et al., 2011) and societal-level quality-of-life indicators (Lambert et al., 2014). Likewise, the possible mental, physical and community health impacts of other forms of environmental disruption are being mapped out; potentially useful are the guidelines on how communities can prepare for the psychological impacts of such disruption (Doherty and Clayton, 2011; Reser and Swim, 2011; Clayton et al., 2014). Similar efforts are needed to prepare individuals and communities for the dramatic yet long-drawn-out social and psychological impacts of energy descent and declining net energy.

Perhaps an affirmative outcome is possible. If handled well, an energy descent could be an opportunity to bring out the best in people (Baker, 2011). This potential was earlier suggested by Hubbert (1996) who analyzed the decline in fossil fuel production rates. Hubbert (1996, p. 126) suggests that if action is taken before the situation becomes unmanageable then, “there is promise that we could be on the threshold of achieving one of the greatest intellectual and cultural advances in human history.” Notice that instead of using wording like stress, disintegration or collapse, Hubbert (1996) envisions a response to biophysical limits as an advance. It is here that the behavioral sciences might be of most use by helping people facing a changing biophysical context to craft new visions of their future, identify new behavior patterns, acquire and share the required skills and motivate the venture. But it is important to start the process while there are still options and surpluses of energy and social capital.

### NOT JUST ANOTHER APPLICATION AREA

Henry David Thoreau famously asked, “What’s the use of a fine house if you haven’t got a tolerable planet to put it on?” (Sanborn, 1894). This same logic—that some things are foundational and must be treated as such—might be applied to the current topic. All through the last century, when cheap energy was available in ever-increasing amounts, the behavioral sciences treated biophysical limits—when thought about at all—as an economic or technological issue more suitably addressed by other disciplines. Such inattentiveness was supported with the widespread assumption that unlimited growth could continue and not adversely affect the earth’s ecosystem—what Daly calls “empty world” thinking (Daly and Farley, 2010). Later, when ecological constraints were finally acknowledged, the behavioral effects and interactions were taken up primarily by sub-disciplines such as environmental and conservation psychology (De Young, 2013). This tendency to specialize the servicing of social needs has been commonplace and generally effective. But it is important to note that the growth and ubiquity of specialization may itself have been made possible by an era of large and predictable surpluses of net energy. Absent those surpluses it may be difficult to continue such an approach.

Stated plainly, helping society to thrive while living within ecological limits should no longer be treated as just another application area of the behavioral sciences. Responding to biophysical constraints has become an existential issue, global in scope, local in impact. Whatever social good can be achieved through the application of empirical discoveries and clinical practices, that good may remain unrealized should society falter in its response to energy descent. Thus, developing a biophysical psychology is an essential pre-condition for attaining the other worthy goals of all social scientists and practitioners.

Ironically, just as it once proved easy to relegate biophysical limits, psychology itself has been ignored by the energy descent community, except occasionally as an instrument for “getting people to behave right.” The real action was argued to be in the physical and policy sciences. Sometimes this dismissal comes from the perspective that since most environmental problems originate from individual behavior (i.e., the sovereign consumer), people cannot be counted on to voluntarily make things right. Other times the negation emerges when behavioral interventions are being compared with other non-behavioral approaches. To present just one example, Newton (2014), in a discussion of energy-efficient neighborhoods, compares technological intervention and voluntary behavior change to sustainable urban design, the latter being that paper’s focus. Despite highlighting the potential for rapid change at low public costs, behavior change is dismissed as being unable to scale-up and spread quickly enough to make a difference. Note, however, that the modifier being used here is voluntary; in the absence of an outside force or precipitating event, so the argument goes, behavior is unlikely to voluntarily change sufficiently. This is a critique that is taken up shortly.

While it is not appropriate to arrogate to the behavioral sciences a special role in forming a response to biophysical limits, it can be noted that, under certain conditions human behavior can innovate quickly and that the behavioral sciences understand those conditions. Furthermore, human nature is not just a source of the problems being faced but also a fount of solutions awaiting dissemination. Indeed, seeking interventions that help craft a better world is a centuries-old quest that cuts across all the social sciences. This would seem to call for broad involvement in forming a biophysical psychology. It echoes the assertion of the American Psychological Association that research addressing climate change cannot be left to just one sub-discipline but must utilize the expertise of researchers and practitioners from multiple areas (Swim et al., 2009). Thus, rather than dismissing behavioral interventions as an ineffective response, many empirical findings from a variety of academic disciplines support the decidedly optimistic view that individual behavior can change in timely, profound and durable ways (Clayton and Myers, 2009; Basu et al., 2014; Kaplan and Basu, in press) and the behavioral sciences know how to help initiate and then support this process of social change.

### SIMPLIFICATION OF BEHAVIOR CHANGE

Early on it was imagined that since more than 70% of what an industrial nation produces is for personal use, encouraging green consumption would be a direct means of achieving environmental sustainability. Thus, behavioral science focused on encouraging green consumerism, a belief that by modifying consumption choices it would be possible to “green and lean” industrial society enough to create an ecological steady-state. This approach drew from the many interventions used to change behavior (De Young, 1993, 2011; Stern, 2000; Abrahamse et al., 2005; Steg and Vlek, 2009; Osbaldiston and Schott, 2012; Steg and Nordlund, 2012). The goal here was to inform, motivate and guide behavior but always with the understanding that behavior change was ultimately voluntary. Rarely was it proposed to directly coerce or restrain consumer choice. Such restrictions, when applied, were usually upstream in the industrial design and production process. Moreover, these restrictions were most often efficiency-driven efforts that affected consumer choice only indirectly (e.g., CAFE standards, EnergyStar appliances) and were usually slow-acting.

Green consumerism is an approach fully compatible with the principle of voluntary behavior change. It poses no threat to business-as-usual within industrial society where consumers are to be treated as sovereign and their purchasing behavior is to remain inviolate (Princen et al., 2002; Princen, 2010). Green consumerism is also a gentle approach because it contains the implicit promise that after achieving a sustainable state, the comforts and conveniences of modernity will remain as would the belief that such a lifestyle could be made available to everyone on the planet.

Unfortunately, despite enlightened efforts at green marketing, environmental education, sustainable design and environmental policy-making, the consumerist life pattern continues to consume the planet. In fact, decades of education and intervention have produced not decline but growth in industrial society’s ecological footprint (Rees, 2010). One could argue that without these interventions, and the behaviors they changed, things now would be much worse. But despite the truth of that statement, humans continue to overshoot the planet’s carrying capacity (Catton, 1982; Turner, 2012) in part because green consumption does not necessarily mean less consumption (Jackson, 2005).

If these prospects are not attention-getting enough, the approaching biophysical limits expose another realization. Underlying the commendable principle of voluntary behavior change is a key supposition. It presumes that circumstances permit people the choice either to continue consuming or to reduce their consumption. Yet, given the biophysical context outlined above, this presumption may no longer be valid. This is what sustainability at its core is about—behaviors that are or become unsustainable will end.

Thus, soon now, whether due to energy descent, declining net energy or some other ecological limit, modern society likely will be consuming less, ready or not. A reduced-consumption existence may become commonplace not because conservation behavior was voluntarily chosen by the public or cleverly initiated by behavioral scientists but because there will be no other choice. Having ignored many opportunities for voluntary simplicity (Gregg, 1936), industrial society now faces involuntary simplicity. It will consume less because there will be less to consume. Dire consequences will still arise from past consumption and its delayed consequences (e.g., drawdown of fossil aquifers, loss of soil fertility, ocean acidification, climate disruption) but future consumption will first slow then decline.

This feature of the new behavioral context, what might be called behavioral simplification, unexpectedly may make the process of transitioning easier. First, the downshift most likely will be slow. As Greer (2012, pp. 97–98) points out, “The resource base of industrial society is shrinking but it’s far from exhausted, the impact of global warming and ecological degradation build slowly over time …” This is not at all what the popular folk mythology of resource apocalypse predicts. It lacks Hollywood’s sudden and catastrophic collapse motif. The change is more likely to emerge slowly over decades—a persistent step-wise downshift to a new normal.

Yet, there are still behavioral challenges to be addressed if this downshift is to be experienced more gently than it might otherwise be. Modern society, as a whole, does not have a settled pattern of voluntarily exploring and adopting alternative life patterns in advance of being forced into so doing. In fact, there is ample evidence that whatever resources were available were consumed to the point of overshoot (Catton, 1982). What behavior change successes there have been all too often are followed by a return to previous consumerist tendencies once the initiating event subsides. Such experiences might leave people with the sense that an effective strategy is to wait out the apparent crisis and anticipate a return to normal.

The rest of this paper recommends an agenda for helping the development of new living patterns before such major adaptations are forced upon society. One issue here involves the choice of how to assess the prospects of responding in time to energy descent. Another is the need to acknowledge that the behavioral and social sciences may not be in charge of the ensuing response. Meadows et al. (2004, p. 284) faced the first issue and framed it as a choice among three prospects:

“… the world faces not a preordained future, but a choice. The choice is between different mental models, which lead logically to different scenarios. One mental model says that this world for all practical purposes has no limits. Choosing that mental model will encourage extractive business as usual and take the human economy even further beyond the limits, the result will be collapse.

Another mental model says that the limits are real and close, and that there is not enough time, and that people cannot be moderate or responsible or compassionate. At least not in time. That model is self-fulfilling. If the world’s people choose to believe it, they will be proven right. The result will be collapse.

A third mental model says that the limits are real and close and in some cases below our current levels of throughput. But there is just enough time, with no time to waste. There is just enough energy, enough material, enough money, enough environmental resilience, and enough human virtue to bring about a planned reduction in the ecological footprint of humankind: a sustainability revolution to a much better world for the vast majority.

That third scenario might very well be wrong. But the evidence we have seen, from world data to global computer models, suggests that it could conceivably be made right. There is no way of knowing for sure, other than to try it.”

Their perspective is a guarded optimism to be sure. But for the issue at hand—shifting behavior patterns to those compatible with biophysical reality—it is particularly fortunate that the patterns that people may need to adopt are not totally without precedent. As Boulding (1978, p. 93) once quipped, “Anything that exists is possible.” The needed patterns are neither new nor untested nor absent from the world at large; what they are is unfamiliar and perhaps unwelcomed by most members of industrial society. But change need not start all-at-once in every corner and with every member of society, indeed it rarely does.

Early experimentation with simple living is recorded in the history of intentional communities in North America (Kanter, 1972; Morris, 2007). A few of these continue to exist, most often as living museums but occasionally as growing communities (e.g., Amish). There also are modern social experiments involving early adopters who are exploring changes to deeply ingrained life patterns. Some are approaches derived from Lewinȉs (1952) pioneering work on using citizen assemblies to affect radical change by first presenting people with the issue being faced and then giving them the trust, time and support needed to develop local responses. A useful update of this method, focused on environmental stewardship, was done by Matthies and Kromker (2000). Several examples, relevant to but not directly addressing energy descent, employ a community-based intervention called EcoTeams (Staats et al., 2004; Nye and Burgess, 2008). These include changing a broad collection of behaviors but center on the household or neighborhood scale and usually focus on consumer behavior.

Some fascinating examples that directly address energy descent and are being implemented at a somewhat larger scale include ecovillages (Liftin, 2011, 2012, 2013a,b) and transition towns (Hopkins, 2008, 2011; Chamberlin, 2009). There is great variation across these settlements but they seem to have in common a focus on environmental and social stewardship. What is fascinating is that these latter explorations were not initiated nor supported by the behavioral or social sciences, corporations, governments or the major non-governmental organizations; they self-initiated. Some of them are being chronicled by scholars but empirical research on their evolution has only just begun (see, for instance^1^).

Perhaps more important than their appeal to the pioneers is the potential for these social experiments to serve as models for other, later adopters. It seems likely that many people will decide to change only after signs of an energy descent become overwhelmingly clear to them. Fortunately for these late adopters, each step down the energy descent, as unnerving as it may be, will likely be followed by relatively stable periods. It may be reasonable to expect the periods in-between each downshift to be stable enough to allow time for exploring and experimenting with alternative life patterns. These intervals also may provide the time needed to build resilience into social and community systems and thus allow these systems to better deal with the next ecological or societal step down (Alexander, 2012).

Although the descent may be slow and punctuated, it likely will be relentless. Early on, each drop may seem like an emergency or crisis making it possible to expect that, with time, things may return to normal. But slowly, every next downshift, coupled with the unknowable duration of each pause, will make such an expectation untenable. Instead, over time, a growing number of people will experience biophysical constraints as unavoidable, directly perceivable and palpable. The need to change behavior would become blatantly obvious. Denial occasionally might be possible, perhaps prompted by the slow descent and stable lulls. But with every next step down an ever growing number of people would find delay to be a non-functional response, maybe even a personally perilous one.

Thus, the long-drawn nature of the descent, with interludes supportive of social experiments, may make behavior change easier. Practitioners and educators may no longer need to persuade people to change but instead would need to be ready to help them to do so. No longer would the public need to judge the veracity of abstract notions of limits-to-growth. Under the signals of an emerging energy descent, the process of societal transition and individual behavior change would not await professional intervention, governmental permission or venture capital support. It would self-initiate—although, perhaps initially, hidden in plain sight.

## PROSPECTING THE COMING TRANSITION

If the biophysical situation and the resulting change in the behavioral context unfold as just outlined then modern society will face an involuntary transition of unprecedented depth and duration. Prefiguring a variety of responses might make for an easier transition. The process of crafting a response may also contain some fascinating opportunities. But getting people to this realization cannot be achieved just by laying out the facts of a potential energy descent. After all, presenting such threats-of-change in the past has rarely prompted environmental stewardship behavior. If that approach had worked as well as needed then society might not be facing the current predicament. And although we may be facing a future of slow regress rather than rapid progress, “regress is quite literally an unthinkable concept these days” (Greer, 2012, p. 99). Thus, taking a direct, facts-first approach has virtually no chance of easing the transition, particularly when the context is not a solvable problem but a complicated predicament.

If we paused for a moment, and reflected on this social dilemma, it might be easy to despair of the human prospect. Yet the issue here may be one of mis-framing and thus lends itself to re-framing. An approach that might have promise is to help people to slowly build their own understanding of the newly emerging biophysical context while simultaneously helping them to explore behaviors that are meaningful to them now, while also pre-familiarizing them with behaviors that might be essential further on.

### THE USEFULNESS OF SMALL EXPERIMENTS

Behavior change under conditions of urgency, great environmental uncertainty and grave stakes might be advised to start with small steps. As anthropologist and political scientist Scott (1998, p. 345) advises with respect to interventions for economic development, “Prefer wherever possible to take a small step, stand back, observe, and then plan the next small move.” Scott’s (1998) suggestion follows, in part, the “small experiment” approach to environmental problem-solving outlined by Kaplan (1996; see also Kaplan et al., 1998; Irvine and Kaplan, 2001). Small experiments are a framework for supporting problem-solving that is based on people’s innate inclinations to explore and understand (Kaplan and Kaplan, 2003, 2009) and on their brain having evolved to prospect the future not just track the past (Seligman et al., 2013). The small experiment approach also supports behavioral innovation, maintains local relevance and experimental validity, all while promoting rapid dissemination of findings. It also contrasts with the large-scale approach that dominates research these days. This framework can help people who are not trained as scientists to discover what works in their locality.

To enhance engagement, the small experiment framework carefully manages the scale of the activity. Picking the appropriate scale is a crucial step. It was Weick’s (1984) insight that people anchor around the scale and structure of the initial problem definition and start to work on responses that are only at that same scale or structure. If we cast the problems faced as being at a large-scale or involving large systems, as is often the case with environmental issues, then it is hard to imagine anything but a large-scale or large system response sufficing. Fortunately, although large-scale problems may seem to demand large-scale responses, the scale of the problem does not dictate the scale of the response.

Small experiments are going on all the time. They are often the basis of stories told by at-home tinkerers, dedicated gardeners, community organizers, and innovative teachers. They are part of team efforts where experts and citizens coordinate and apply their talents and knowledge to an issue of mutual concern. Consider the many pilot programs, demonstration sites, field tests, and trial runs regularly reported in both popular and scientific publications, as well as the neighborhood, community, and village examples mentioned earlier. In fact, small experiments are so common that they may seem inconsequential to the casual observer; nevertheless they can be a powerful means of behavioral entrepreneurship. Despite their ubiquity, Kaplan (1996) and Irvine and Kaplan (2001) have discussed guidelines for enhancing the effectiveness of small experiments and broadening their appeal beyond just early adopters. Very briefly summarized, these are:

#### Scale

While already an integral aspect of small experiments, smallness can be understood in a variety of ways. Keeping the physical scope small is obvious. Others include keeping the time-span short and the breadth of exploring restricted as well as involving only a small number of people. The experiment can also be tentative, tried out for a limited time. These guidelines help keep the costs of project initiation and management low.

#### Expectations

So too should expectations be kept in check. The findings of small experiments are unavoidably imperfect and incomplete. Yet small too are the consequences of failure; failure is always a possibility if an experiment is genuine. Even so, findings from a modest enterprise may prove extraordinarily useful and have broad effects.

#### Goal and focus

An energy descent would be felt in all parts of society and affect all daily activities. Nevertheless, it is useful to keep the focus of the small experiment on only one specific issue. While it may be okay to start exploring before having everything in place, it is essential to first have a concise question. Anticipating what would be most useful to be able to say at the end is an excellent way of formulating the initial question.

#### Tracking and record keeping

Empirical research, at its core, involves being attentive to what is going on. Whether formal or informal information gathering is used, the objective is to systematically learn what worked and what did not. In the immediate timeframe and at the local level, the tracking allows for feedback to those directly engaged. Over a longer timeframe, it informs next steps and may provide the basis for developing generalizations that might be useful elsewhere.

#### Dissemination and communication

Sharing the successes of a small experiment is a way to let the people involved knows that their efforts matter. It is also an opportunity to validate the correctness of the proposed changes for the community members who were not engaged in the effort. Finally, communicating with people at a distance may provide credibility to other small experiments and help to motivate and support the efforts of later adopters; successes in one locality become plausible options to explore elsewhere, while communicating about failures instills caution and may prevent wasted effort.

It is noteworthy that nothing in these guidelines restricts small experiments to taking only small steps or to a slow discovery process. A behavior change process called adaptive muddling stresses this subtle but important issue and also adds the element of stability to the small experiment framework (De Young and Kaplan, 1988). A stability component is used to reduce the cost of failure for the individuals involved. It also makes highly improbable unchecked and disorienting social change. With a safety net in place people need not privilege the status quo by investigating only marginal behavior change. Far reaching change can be contemplated, explored and tentatively adopted. The scale of the experiment may be small but adaptive muddling supports people exploring life-changing responses to the advent of biophysical limits.

The small experiment notion also provides researchers with a framework for exploring a number of behavioral questions with important practical implications. Among the questions that seem to me most urgent to explore are the following: what are the conditions under which people domesticate the notion of a dramatic biophysical descent? Does the new behavioral context require a shift from green consumerism to green citizenship? Are there intrinsic reward embedded in crafting a response that may hasten the process? Might it be possible that crafting a response to energy descent directly increases well-being? Each is briefly outlined below in the hope that they inspire future work or, where sufficient research exists to fully address the question, package known science as guidelines to “give away” to practitioners. Clearly, this list is not exhaustive. Researchable questions about the behavioral aspects of energy descent abound and span all of the social sciences.

### PRE-FAMILIARIZATION

In conversations about behavior, it is often claimed that people resist change because they rigidly anchor to the status quo. Also referred to as behavioral inertia, this tendency has been identified as a major impediment in efforts to get people and institutions to adapt to global environmental change (World Bank, 2009). The power of the current situation is related to cognitive availability (Tversky and Kahneman, 1973). The instances or examples that can most readily be thought of, those that are most mentally available, become powerful predictors of the things to which people attend and that motivate them. Thus, it would seem that the existing state of the world is what is both justified and deemed desirable (Jost et al., 2004). Under this framing, crafting a response to an energy descent would be heavily burdened by the collective, tangible and century-long experience of exponential growth in available energy, material consumption and consumer sovereignty.

However, an alternative perspective is to reframe what might appear to be a bias toward the status quo as, instead, a bias toward the familiar (Kaplan and Kaplan, 1983). Although this may sound as if it is an insignificant, perhaps even an academic distinction, it may be one with major implications for adapting to the coming downshift. A status quo bias means that very little can change, and what does change will stay close to present circumstances. A familiarity bias, in contrast, means that change is limited not by where people are now but is also affected by what they know and where they imagine themselves going.

Clearly, adapting to a long-drawn decline in resource availability is not something with which people are currently familiar. We can know that humans once lived this way, and that many still do, but most citizens of industrial society do not. The researchable question here is how such familiarity might be influenced. Does a bias for the familiar always lead people to favor the more physically tangible status quo rather than an imagined future reality, thus identifying this bias as a barrier for transitioning to a new behavior pattern? Or can tangible imagery create knowledge vivid enough to be a sufficient substitute for direct experience (De Young and Monroe, 1996; Kaplan, 1972; Hunt, 1984)? The challenge here is to discover if there are conditions under which knowledge of an emerging but not yet present energy descent, coupled with people’s future aspirations, creates a familiarity as powerful as that created by people’s current circumstances.

It might be fascinating to examine the role and successes of the arts and humanities in pre-familiarization. Kaplan (1972) points to the influence on the behavior of medieval society exerted by the anticipation of hell. Society needs far more useful examples than the existing melodramatic tales of techno-expansion (e.g., the Star Trek franchise) or eco-collapse (e.g., Hunger Games, The Road). Liftin (2011) has done this in her inspiring video narrative of the ecovillage movement. Similar efforts at affirmative storytelling exist at the Resilience^2^ and Transition Network websites^3^. But missing, yet needed, are examples from a behavioral science perspective. The goal would be to share stories that not only honestly portray life under a prolonged and involuntary energy descent, but do so in a way that people crave the experience enough to seek it now.

### GREEN CITIZENSHIP

Another way to understand the behavioral aspects of energy descent is to ask what social role people may need to play. Traditionally, in efforts to promote conservation behavior, people have been cast in the role of green consumer. The researchable question here is to ascertain if this role—once thought adequate for the creation of a sustainable society—is now insufficient to the task of responding to a long-drawn-out energy descent. Then, if the circumstances being imagined here demand that each of us take up green citizenship, how might this new role be promoted?

The distinction between these roles is subtle. Citizens likely have long-term intentions, motives and sources of social-support that differ from and are broader than those of consumers. These differences might constitute opportunities for behavior change. For instance, if the changing biophysical context can be conveyed in a way that does not frighten or overwhelm people, then we may be able to leverage considerable behavioral entrepreneurship.

But promoting green citizenship over green consumerism may require altered forms and formats of behavioral interventions. Yet, to date, almost all attention, funding, and science has been focused on the green consumer. The few exceptions suggest that green citizenship is a far more complex role than just selecting from among the green products in the marketplace (Gilg et al., 2005; Evans and Abrahamse, 2009). The related concept of ecological citizenship is also being explored (Dobson, 2003, 2006, 2007; Wolf et al., 2009; Wolf, 2011). It provides a starting point for answering the question posed here but it may need to be expanded to both fit the changing biophysical context and accommodate the broader array of motives that promote green citizenship. Of particular importance may be adding the requirement of coping with energy descent while having diminishing amounts of the infrastructure and capital inherent in industrial civilization. Furthermore, ecological citizenship presupposes that a deep attitude change precedes any significant behavior change. However, the behavioral response to energy descent may be driven as much by necessity as by a prior change in attitude, although the latter may hasten developing a response.

### EMBEDDED BENEFITS

It is possible to imagine three broadly framed categories of behavior necessary to thrive through a prolonged downshift. The first is a response to the unsustainability of hyper-specialization. Individuals and their communities will need to become adept at many new or newly relearned skills. Knowledge and abilities either long dismissed as outdated or consigned to the ranks of the working-class may once again be widely needed. Rather than being efficient in narrow domains, people may need to become proficient at many crafts, have broad practical knowledge, retain the capacity to mindfully plan and restrain their behavior, and be willing to continuously build and share these competencies.

People will also need to be resourceful in ways true to the original definition of that word. Society will come to value the ability to deal with a difficult situation while utilizing only those resources currently at its disposal. Equally respected will be practical knowledge of how to work with the natural world in the thriftiest of ways. This is frugality, an ability that will need to become widespread if a community is to prosper.

Finally, there will be a need for people to maintain their pro-social inclinations and develop those character strengths that make them welcome as community members. For some people this will mean the ability to take and hold a leadership role. For everyone it will mean remaining cooperative through lean times and exhibiting forbearance and genuine kindness while under stress. These are attributes that will help people and their community to flourish.

Pursuing these behaviors likely will make it easier to transition through an energy downshift. The behavioral simplification mentioned earlier may go a long way in motivating people to pursue these actions since at some future time, in order to thrive, people will realize that there is no other option. But to prevent a last-minute, hasty, crisis response to the changing biophysical context, it would be necessary to begin this transition before circumstances demand it of us. As Liftin (2012) suggests, society would be better off if it begins prefiguring viable alternative life patterns in the midst of current circumstances.

Putting aside the existential benefit of starting the transition sooner rather than later, the challenges here are significant. Currently, except for a small subset of the population in industrial societies, the behaviors being called for are not those toward which people typically strive. Furthermore, the more common set of motives guiding the behavior of citizens of western society seem unlikely to initiate or sustain the behavior change that eventually will be needed. So, for instance, the need for frugality just mentioned may be valued in the abstract by people and eventually be necessary for community survival, yet it is presently practiced by very few individuals within industrial society. In fact, this once commonplace virtue (Nash, 1998) has become much maligned and dismissed as old-fashioned. It seems likely that any motivation to be frugal is presently overwhelmed by the motivation to be comfortable, to be successful or to better our family’s standard-of-living. Thus the researchable question here is to determine if it is possible to motivate the needed behavior change in advance of any circumstances that demand it of us, and if that is possible, identify the procedures needed to do so.

The first part of the question, the possibility of initiating enduring behavior change, has been well explored. There is evidence that humans are capable of being intrinsically motivated to pursue the abilities mentioned above (Max-Neef, 1992; O’Brien and Wolf, 2010; Sheldon et al., 2011; Howell, 2013; Van der Werff et al., 2013). Chawla (1998) found that environmentally involved individuals credit intrinsic motivation when explaining their sense of integrity in living up to deep values and the competence they feel in both responding to difficulties and from interacting effectively with others. Crompton (2008) reports that, when compared to extrinsically motivated behavior, being intrinsically motivated leads to greater behavioral intensity and perseverance. Respondents in a study done by Wolf (2011) indicated that they derived substantial intrinsic satisfaction from pursuing behavior patterns that address environmental disruption and Brown and Kasser (2005) found that intrinsically oriented individuals engaged in a wider variety of conservation behaviors than did other respondents. In a series of small studies respondents from industrial societies reported deriving deep and direct intrinsic satisfactions from just the sort of behaviors that will be needed for a robust response to energy descent (De Young, 1996, 2000, 2012).

This brings us to the second part of the question—the need to develop and share procedures for using intrinsic motivation. What complicates the matter is that these motives can only be highlighted for people—they can be supported and leveraged but direct manipulation of an intrinsic motive is an oxymoron. The field of self-determination theory has pursued this issue for some decades (Deci and Ryan, 1985, 2012; Ryan and Deci, 2000) and has recently applied its findings to conservation behavior change (Osbaldiston and Sheldon, 2003; Sheldon et al., 2011). But a great deal more research is needed, particularly work that packages theoretical and empirical findings as guidelines for practitioners engaged in social transitions.

### BIOPHYSICAL LIMITS, PSYCHOLOGICAL ABUNDANCE

A provocative suggestion is that a resource downshift has the potential to support, perhaps even to increase, well-being (Illich, 1974; Astyk, 2008). This notion might astonish members of industrial society where material deprivation is seen only as a source of suffering. The more common assumption about causality is in the opposite direction: interventions create conditions that enhance well-being which then allows people to build the personal resources needed to later act in sustainable and generative ways. For instance, Carter (2011) discusses how cultivating positive emotions, with the intention of thereby enhancing the personal resource categories of mindfulness, self-efficacy and positive relations with others, subsequently inspires environmentally responsible behaviors.

The researchable question here is whether it is possible that the effect also functions in the other direction, whereby enhanced well-being is derived from responding well to energy descent. There is some support emerging for this perspective: there are reports of positive interactions between the pursuit of sustainable behavior and the derived well-being elements of enhanced happiness and satisfaction (Corral-Verdugo et al., 2011a,b). Other researchers are exploring the interaction between the pursuit of conservation behavior and sustainable well-being (O’Brien, 2008; Kasser, 2009; Kjell, 2011). Brown and Kasser (2005) report that voluntarily reducing material consumption can be a direct source of subjective well-being (although it will be important to learn if this conservation-behavior-derived well-being also occurs under the involuntary reduction that biophysical limits may cause). Corral-Verdugo (2012) integrates these findings into a proposal for a positive psychology of sustainability where conservation behavior is viewed as the result of deliberation but is maintained by perseverance and derived satisfactions and where pursuing a sustainable life pattern enhances mental health.

However, environmental stewardship behavior may have different effects on two major categories of well-being (i.e., eudaimonia, or meaning-driven well-being versus hedonia, or pleasure-driven well-being). In particular, it is argued that there is a strong positive relationship between sustainability behavior and eudaimonic well-being (Myers, 2003; Jackson, 2005; Kasser, 2009). Venhoeven et al. (2013) report that pro-environmental behavior itself can be experienced as meaningful action that directly increases eudaimonic well-being, a suggestion that is consistent with the embedded benefits concept discussed above.

The possibility that pro-environmental behavior may increase eudaimonic well-being is made all the more significant by a recent discovery that pursuing different types of well-being has a differential effect on the human stress response and immune system functioning. Fredrickson et al. (2013) report that being in a state of hedonic well-being produces an undesirable elevation of inflammatory gene expression, while experiencing eudaimonic well-being causes a beneficial up-regulation of antibody synthesis genes and a down-regulation of pro-inflammatory genes. Thus, it sometimes may be ill advised to focus on the pleasant natural consequences of a behavior or to add pleasurable aspects to those behaviors lacking them. From the perspective of physiological stress and immune system functioning, the lack of inherent pleasure in the transition process may be far less important than people being able to frame the tasks involved as being consequential in some larger context. Thus, if handled well, the difficulty in responding to biophysical limits may, quite unexpectedly, be its redeeming quality.

Yet, in industrial society eudaimonic well-being is often trumped by the pursuit of hedonic pleasure. It is a researchable question whether the promise of eudaimonic well-being derived from responding well to a drawn-out energy descent can be made to overcome the hedonic pleasure gained from pursuing business-as-usual for as long as it lasts. This raises another confounding issue to be dealt with. Meaningful behavior can contribute to eudaimonic well-being, yet to have that effect it seems crucial that the choice to act be autonomously initiated (Ryan and Deci, 2000; Ryan et al., 2008; Venhoeven et al., 2013). External coercion or temptation of any sort or from any source to pursue a sustainable pattern-of-living might preclude any gain in eudaimonic well-being (Evans and Jackson, 2008) and thus negate any subsequent health benefits. As Venhoeven et al. (2013, p. 1379) point out, “it is more likely that only those who deliberately choose a pro-environmental lifestyle will gain eudaimonic well-being from their engagement.”

This poses a dilemma since under an energy descent scenario the need to respond could hardly be thought of as autonomously chosen. If the ultimate goal is to encourage durable behavior change while also enhancing well-being and health then it would be counterproductive to force people to relearn lost skills, pre-familiarize themselves with simple living patterns or adopt conservation behaviors. The researchable question here is whether it is possible to support autonomous motivation under conditions of immutable biophysical limits, and how to craft interventions that create, but not coerce, pre-familiarization.

Yet, despite the need for more research, psychology does have advice to offer. It has been known for some time that effective functioning benefits from an environment responsive to one’s attempts to function (Carr and Lynch, 1968). An environment is responsive when it allows people to experiment and to tentatively try out new ideas even under the pressure of time and resource constraints (i.e., limits-to-growth). An environment that is open and supportive provides a setting where people can more easily learn for themselves. In a responsive environment people can discover how the world functions and what sorts of plans and intentions fit the new biophysical circumstances. Knowing what is and what is not achievable helps to focus one’s attention on those areas that have the greater effect. This brings the discussion back to small experiments which become all the more important when the biophysical and behavioral context has changed from what people are familiar with.

## CONCLUSION

It may seem that much of human behavior is at odds with living within biophysical limits. We have clearly overestimated the capacity of the planet to provide for growth of all kinds, to secure material well-being and to absorb the waste of industrial society. It seems that few members of that society feel any sense of urgency in making these things right. Interventions to change this state of affairs have not had the needed effect. This assessment has brought pause to the environmental and conservation psychology community.

How we respond to the coming age of biophysical limits is one of the defining questions of our time. Yet, there are several reasons why this is a difficult topic to discuss. First, if the biophysical scenario happens anywhere near as outlined above, then modern civilization is facing major changes for which it is currently unprepared. Second, it is being suggested that the behavioral sciences have been ignoring the implications of this scenario. In fact, it may be difficult to take up the challenge of responding to ecological limits since taking that step could be construed as abandoning other cherished social goals, the pursuit of which might depend on the largess of industrial society. Of course, a reassessment of the changing biophysical context suggests that industrial society soon may be hard pressed to continue supporting our pursuit of those valued objectives, thus calling for a realignment of our approach. Finally, a tenet of environmental communication is to emphasize only the positive and to highlight success stories, to do otherwise is to risk losing the audience. However, if society is facing a challenge as daunting as that discussed here then it deserves to be told the effects of reaching biophysical limits and the costs of ignoring those limits.

Society still has options. There is a great deal that the behavioral sciences can do to ease the coming transition. The task, if we are willing to take it up, is to help people cope with the realization that everyday life may soon differ substantially from conventional expectations and to help them envision an alternative to their current relationship with resources. Acknowledging the biophysical trends is the sobering part. Next comes the hopeful part, indeed the exciting part.

## Conflict of Interest Statement

The author declares that the research was conducted in the absence of any commercial or financial relationships that could be construed as a potential conflict of interest.

## Footnotes

1 www.transitionemergingstudy.ca/

2 www.resilience.org

3 www.transitionnetwork.org
